# Regulatory Monitoring of Fortified Foods: Identifying Barriers and Good Practices

**DOI:** 10.9745/GHSP-D-15-00171

**Published:** 2015-09-02

**Authors:** Corey L Luthringer, Laura A Rowe, Marieke Vossenaar, Greg S Garrett

**Affiliations:** ^a^​ Global Alliance for Improved Nutrition, Large Scale Food Fortification, Geneva, Switzerland; ^b^​ Project Healthy Children, Cambridge, Massachusetts, USA

## Abstract

While fortification of staple foods and condiments has gained enormous global traction, poor performance persists throughout many aspects of implementation, most notably around the critical element of regulatory monitoring, which is essential for ensuring foods meet national fortification standards. Where coverage of fortified foods is high, limited nutritional impact of fortification programs largely exists due to regulatory monitoring that insufficiently identifies and holds producers accountable for underfortified products. Based on quality assurance data from 20 national fortification programs in 12 countries, we estimate that less than half of the samples are adequately fortified against relevant national standards. In this paper, we outline key findings from a literature review, key informant interviews with 11 fortification experts, and semi-quantitative surveys with 39 individuals from regulatory agencies and the food fortification industry in 17 countries on the perceived effectiveness of regulatory monitoring systems and barriers to compliance against national fortification standards. Findings highlight that regulatory agencies and industry disagree on the value that enforcement mechanisms have in ensuring compliance against standards. Perceived political risk of enforcement and poorly resourced inspectorate capacity appear to adversely reinforce each other within an environment of unclear legislation to create a major hurdle for improving overall compliance of fortification programs against national standards. Budget constraints affect the ability of regulatory agencies to create a well-trained inspector cadre and improve the detection and enforcement of non-compliant and underfortified products. Recommendations to improve fortification compliance include improving technical capacity; ensuring sustained leadership, accountability, and funding in both the private and the public sectors; and removing political barriers to ensure consistent detection of underfortified products and enforcement of applicable fortification standards. Only by taking concrete steps to improve the entire regulatory system that is built on a cooperative working relationship between regulatory agencies and food producers will a nutrition strategy that uses fortification see its intended health effects.

## Abstract (precis)

Food fortification with micronutrients often is not compliant with relevant standards, in large part because poor regulatory monitoring does not sufficiently identify and hold producers accountable for underfortified products. We propose these reinforcing approaches: clear legislation, government leadership, strong enforcement of regulations, improved financial and human capacity at the regulatory agency and industry levels, civil society engagement, simplified monitoring processes, and relationship building between industry and government.

## INTRODUCTION

Large-scale food fortification is widely recognized as a cost-effective strategy to improve the micronutrient status of populations,^1^^-^^3^ and it has been linked to economic benefits resulting from improved productivity, increased earnings potential, and GDP growth.^4^^-^^8^ As part of a comprehensive approach to support increased intake of critical micronutrients, fortification is a highly sustainable intervention when properly applied and regulated.^9^^,^^10^ Fortification will increasingly become more relevant as food industries consolidate and penetrate markets in rural areas while at the same time populations urbanize and increase their consumption of centrally processed foods.^11^^,^^12^

Fortification has gained global traction, especially in middle-income countries. Governments, industry, and civil society have come together to implement salt iodization programs in more than 140 countries worldwide^13^; 83 countries have mandated at least one kind of cereal grain fortification,^14^ 23 countries have mandated fortification of edible oils,^15^ and nearly a dozen countries fortify condiments. According to the Food Fortification Initiative and the Iodine Global Network, 31% of commercially milled wheat flour is fortified, reaching more than 2 billion people, and 76% of households are consuming iodized salt.^13^^,^^14^^,^^16^

Despite progress, there exists evidence of non-fortification and underfortification among products claiming to be fortified. Among mandatory fortification programs in low- and middle-income countries in Africa and Asia, fortification coverage and compliance can be triangulated from a variety of data sources. From household coverage data, available for 10 national salt iodization programs, a population-weighted average of 50% of households have access to adequately iodized salt.^17^ From industry self-reported quality assurance and quality control (QA/QC) results from national fortification programs in 5 countries, representing 2 maize flour, 5 wheat flour, 1 sugar, and 4 vegetable oil programs, it is estimated that 45% of product samples are adequately fortified per national standards.^18^^,^^19^ Accelerated degradation of certain fortificants has been seen in some climates, leading to products being underfortified when tested. These cases can and should be controlled by improving overall QA/QC practices and storage conditions by industry. This can be successfully undertaken by industry when strong regulatory monitoring practices are in place.^20^^,^^21^

Of households with access to fortified foods, less than half are consuming adequately fortified foods, according to data from 22 national fortification programs.

**FIGURE 1:**
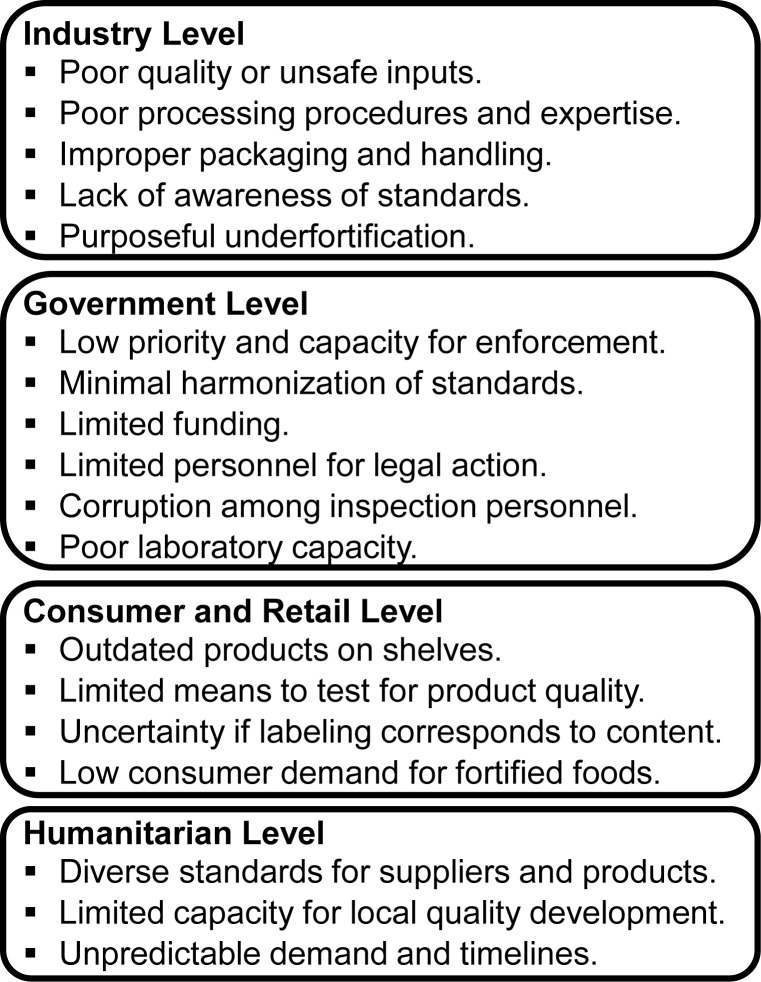
Critical Challenges Along the Food Value Chain That Present Barriers to Consistent Compliance Against National Fortification Standards

Evidence of underfortification confirms the slow progress in improving compliance against fortification standards. Identified challenges at industry, government, and retail levels suggest critical points along the food value chain that prevent consistent compliance (Figure 1). Challenges also exist at the humanitarian level since food-related assistance often occurs in parallel with national fortification initiatives and can present barriers to compliance. Good regulatory monitoring and QA/QC practices can help ensure that food products advertised as, or required by law to be, fortified are *adequately* fortified, meaning compliant with relevant regulations or fortified as claimed. There is limited value in measuring the health impact of fortification programs if fortified foods are not adequately fortified with a bioavailable fortificant and government regulatory monitoring systems are unable to detect underfortified products and hold their producers accountable.^20^^,^^22^^,^^23^ Ensuring adequate fortification is necessary to improve micronutrient consumption via national fortification programs.

Regulatory monitoring is the continuous collection and review of information at key delivery points to ensure fortified foods meet national standards.^21^ Regulatory monitoring encompasses internal control, including QA/QC activities that are the responsibility of the food producer, and external monitoring, including inspections and audits that are the responsibility of government authorities.^24^ The purpose of internal control is to identify and remedy irregularities throughout the production and packaging processes. Governments use external monitoring to verify that manufacturing steps are properly implemented and result in a quality product. External monitoring by governments should *complement*, not replace, QA/QC processes and tests related to fortification at the production level.^24^

Regulatory monitoring of food fortification includes internal quality assurance and control measures as well as external monitoring by government.

Many documents outline key elements required for successful fortification programs. These requirements focus mainly on technical, infrastructural, and socioeconomic constraints that affect the supply and demand of fortified foods; they do not focus on what is needed for effective regulatory monitoring.^24^^,^^25^ Several manuals discuss regulatory monitoring of fortified foods; however, they focus on ideal QA/QC processes and product sampling and testing methods at the production site.^22^^,^^24^^,^^26^^-^^28^

The legal framework specific to food fortification provides the basis for ensuring product quality, safety, and the achievement of public health nutrition goals. Legislation should provide the basis for external monitoring systems, including clear delineations of stakeholder roles and responsibilities and a prescription of enforcement tools to deter non-compliance.^29^^-^^31^ There is little practical guidance in the literature beyond the rationale and theory underpinning external monitoring of food fortification programs.

Many governments struggle to identify good practices in regulatory monitoring, including their role in external monitoring and in supporting industry to improve their internal control. There is insufficient practical knowledge on elements of effective and efficient external monitoring systems and a clear need to communicate lessons learned in this area. This study intends to fill this gap by providing a qualitative assessment of the barriers and successes experienced by regulatory monitoring systems and industries that fortify in low- and middle-income African and Asian countries and their perspectives on factors that contribute toward regulatory monitoring effectiveness.

## METHODS

The World Health Organization (WHO) has developed guidelines on fortification that describe key functions of regulatory monitoring and that identify criteria for evaluating monitoring systems.^24^ These criteria include having an established set of procedures, methodologies, and reporting requirements to continuously assess the fortification program; a clear delineation of responsibilities; and an efficient feedback mechanism that facilitates the implementation of corrective measures.^24^ These are corroborated by similar criteria identified in the FORTIMAS approach for tracking fortification impact.^32^ This study builds on these criteria by asking individuals involved in regulatory monitoring to reflect upon their successes, and it challenges them to create a picture of what effective monitoring would look like in their country context. This was done through a literature review, key informant interviews, and a semi-quantitative questionnaire.

A desk review of gray and published literature was conducted from the authors’ personal libraries and a PubMed search on keywords of “regulatory monitoring” or “regulatory compliance” as it relates to “food and drug manufacturing” or “food fortification.” Key informant interviews were conducted with experts from fortification project personnel and public and private entities involved in fortification. The literature review and interview results were used to generate key themes, learn success stories, and develop 2 structured, semi-quantitative questionnaires on elements of regulatory monitoring within a mandatory fortification environment. One questionnaire was designed for respondents from government regulatory agencies and the other for respondents from industrial corporations that fortify a staple food, with overlapping questions and themes. The questionnaires were designed to elicit responses in each of the 5 major components of food control as designated by the Food and Agricultural Organization (FAO) and WHO^29^:

- Food law and regulations
- Food management
- Inspection services
- Lab services
- Information, education, communication, and training

While many food producers in low- and middle-income countries in Africa and Asia lack adequate QA/QC and good manufacturing practices, certainly contributing to persistently underfortified foods reaching consumers, the questionnaire focused on external monitoring and the role of government regulatory agencies.

Questionnaires were deployed to contacts in regulatory agencies and industry in 28 countries with a focus on low- and middle-income countries in Africa and Asia.^33^ Snowball sampling was employed, asking respondents to suggest others in their networks who would qualify for participation. Questionnaires were administered via an online survey available in both English and French. Country staff from the Global Alliance for Improved Nutrition (GAIN) and Project Healthy Children (PHC) were encouraged to work with respondents in a structured interview format to translate and facilitate question understanding.

In total, 55 respondents participated in the questionnaire; 39 (71%) were included in the analysis. Inclusion criteria for the analysis comprised answering a set of key questions (9 responses excluded) and, in the case of industry respondents, a requirement that the food vehicle fortified on site was included in the country’s mandatory fortification legislation (4 responses excluded). In all but 2 cases, exclusion due to non-completion was due to technical and Internet connectivity issues; a subsequent questionnaire was completed from these countries as a second attempt with the online survey tool or via an identical paper version.

Analyses were conducted at the respondent level. Some countries had multiple respondents; however, the lack of agreement between respondents within the same country and sector as well as the nature of the study design led to the choice not to average and weight responses or analyze data at the country level. Additionally, it was not the aim of this study to compare progress and practices across countries but to gain a sense of respondents’ perspectives on regulatory monitoring effectiveness, barriers, and best practices.

## FINDINGS

Key informant interviews were conducted with 11 fortification experts from program implementers, industry, and government. Questionnaire responses were completed by 18 individuals from regulatory agencies in 15 countries and by 21 individuals from the food fortification industry in 13 countries. A total of 17 countries were represented. Eleven countries provided at least 1 completed response from both the regulatory agency and industry (Afghanistan, Bangladesh, Ethiopia, Ghana, Indonesia, Kenya, Nigeria, Pakistan, the Philippines, Senegal, and Tajikistan). Two countries provided respondents from industry only (Egypt and Kazakhstan) and 4 countries provided respondents from regulatory agencies only (Kyrgyzstan, Liberia, Mozambique, and Nepal).

As reported by regulatory agencies across the 17 countries at the time of the survey, every country mandates salt iodization. Eight countries also mandate fortification of wheat flour, and 9 countries mandate fortification of vegetable oil. Four countries mandate the fortification of 4 or more vehicles (salt plus some combination of wheat flour, maize flour, rice, sugar, and vegetable oil). Thirteen industry respondents fortify salt at their facilities, 6 fortify wheat flour, 2 fortify vegetable oil, and 1 fortifies maize flour. Industry respondents indicated they began iodizing salt between 1991 and 2009, while the fortification of other vehicles began between 2000 and 2013.

**FIGURE 2:**
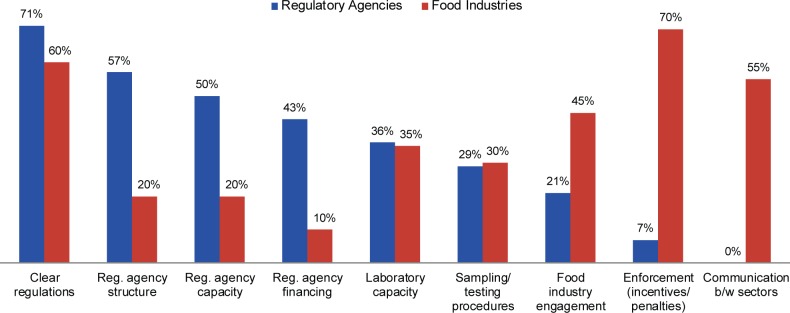
Top Regulatory Monitoring Priorities Requiring Improvements at the Regulatory Agency Level to Ensure Industry Compliance With Fortification, According to Rankings^a^ by Questionnaire Respondents From Regulatory Agencies (n=14) and Food Industries (n=20)^b^ ^a^ Respondents ranked each element as 1 of their top 3 priorities. ^b^ The 14 respondents from regulatory agencies came from 12 countries, and the 20 respondents from food industries came from 13 countries (a total of 16 countries represented). Four respondents from regulatory agencies and one from industry left this question blank.

Survey respondents prioritized a list of regulatory monitoring elements needing improvements at the regulatory agency level to ensure compliance against relevant national fortification standards (Figure 2). Respondents from both regulatory agencies and industry placed a high importance on improving regulations so they are clear and provide a good regulatory environment including delineating the roles and responsibilities of stakeholders. However, regulatory agencies and industry inversely prioritized the remaining elements: Industry respondents rated incentives and penalties for enforcement, communication between sectors, and industry engagement as their next highest priorities for regulatory agency improvement, whereas these components were among the lowest priorities for regulatory agencies.

Both regulatory agency and industry respondents placed high importance on improving clarity of regulations.

**FIGURE 3:**
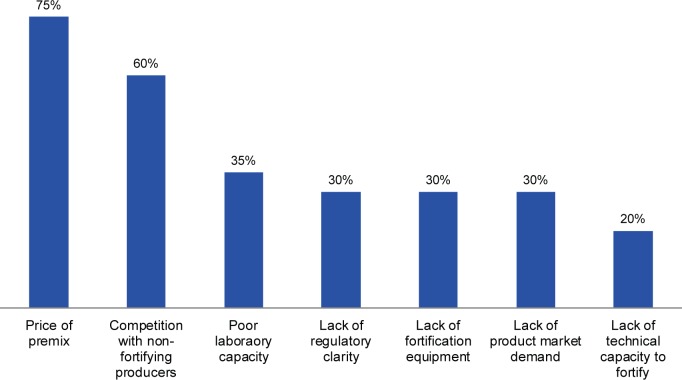
Top Barriers^a^ Fortified Food Producers Face in Ensuring Fortification Compliance, According to Rankings by Questionnaire Respondents From Food Industries (n=20)^b^ ^a^ Respondents ranked each element as 1 of their top 3 barriers. ^b^ The 20 respondents came from 13 countries; 1 respondent left this question blank.

Industry respondents were asked to prioritize a list of barriers they and other producers of fortified foods face in ensuring adequate fortification (Figure 3). Two barriers dominated the list perceived by the food industry: the high price of premix (the powdery blend of vitamins and minerals used in fortification), which drives up the cost of processing, and competition with non-fortifying or non-compliant producers, which illustrates the need for mandatory legislation and enforcement to level the playing field.

Based on the perceived importance of each element and the identified barriers, 3 main themes were chosen for further investigation during analysis: (1) food law and legislative environment, (2) mechanisms of regulation enforcement, and (3) prioritized human and financial resources at regulatory agencies.

### Food Law and Legislative Environment

**FIGURE 4:**
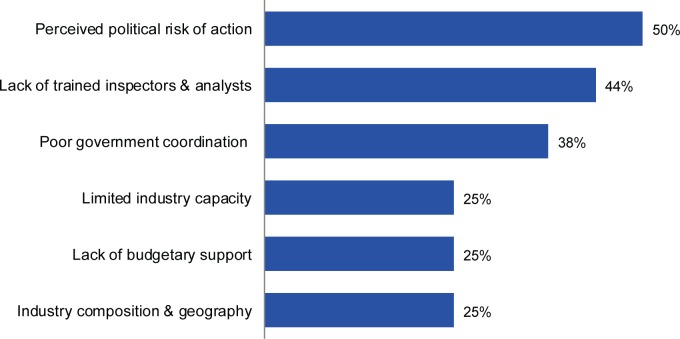
Greatest Challenges Faced in Creating a Legal and Regulatory Environment That Allows for High Compliance With Fortification Regulations, According to Open-Ended Responses From Regulatory Agency Respondents (n=16)^a^ ^a^ The 16 respondents came from 13 countries, with some respondents providing multiple answers; 2 respondents left this question blank.

The lack of clarity in the roles of government authorities in monitoring fortified foods was a barrier faced by 6 of 20 industry respondents (30%), while 22 of 34 respondents from both sectors (65%) identified the need for clear regulations as a top priority for fortification compliance. Figure 4 depicts the challenges regulatory agencies reported facing in creating a legislative environment conducive to fortification compliance. Half of the respondents perceived a political risk to taking regulatory action, and 44% indicated a lack of trained inspectors and analysts as a key challenge.

Half of regulatory agency respondents perceived a political risk to taking regulatory action against the food industry.

Legislative instruments should be robust enough to prevent risks to safety and quality while also flexible enough to allow for changing technology and local nutrition contexts. Fifteen of 39 respondents (38%) considered their country’s regulatory system responsive to new technologies; 3 respondents (8%) considered it very responsive (data not shown).

Technical regulations are delineations of a product’s characteristics, such as size, shape, or performance, which are mandatory for producers to conform to. In this way, they are legally enforceable and can be more easily modified without having to pass entirely new laws. One questionnaire respondent stated that using technical regulations *“… made the standard a mandatory tool without having to go to Parliament for a new legislative instrument.”*

### Mechanisms of Regulation Enforcement

Key informant interviews with government and industry stakeholders opined that economic incentives send a strong message to the private sector that the government will share in the risks and rewards of fortification and make a financial commitment. As one questionnaire respondent described, *“Compliant industries are recognized publicly, and messages about the purpose of enforcement are regularly communicated, helping to gain industry buy-in.”*

Among regulatory agency questionnaire respondents, 14 of 17 (82%) reported deploying an incentive, and 11 of 20 from food industries (55%) reported that an incentive had been used to sway their behavior toward compliance against national fortification standards. Positive brand naming was reportedly used most often by regulatory agencies, followed by subsidies for inputs such as premix or equipment. Respondents from both sectors believed that incentives, as they are currently used, could improve in their effectiveness in encouraging compliance with mandatory fortification legislation; 8 of the 27 respondents (30%) reported incentives are “very” effective, while 9 (33%) considered incentives “moderately” effective, and 10 (37%) considered them “slightly” or “not at all” effective.

Both regulatory agency and industry respondents believed that incentives could encourage compliance with fortification regulations.

Over 90% of questionnaire respondents (34 of 37) reported that penalties have been used to deter non-compliance, although 28 of 34 (82%) admitted inconsistent use or ineffective enforcement of penalties. Fines, operating license suspension, and factory closure were reportedly used most often. Similar to their views on incentives, of 34 respondents, 10 respondents from both sectors (29%) viewed current use of penalties as “very” effective, while 15 (44%) considered penalties “moderately” effective, and 9 (26%) reported penalties are “slightly” or “not at all” effective.

A recurrent theme from key informant interviews and questionnaires was a perceived political risk surrounding consistent enforcement. Eleven of 18 respondents from regulatory agencies (61%) believed this lack of willingness to take on the political risk of enforcement is a major barrier. Regulatory agencies claimed using penalties is politically risky due to perceived or real resistance from interest groups. One respondent described it as risky *“… for political reasons, because of backlash and strike threat by the mill association.”*

Respondents opined that the political risk of enforcement leads to penalties that are not severe enough to encourage adequate fortification or are inconsistently applied due to insufficient resources required to navigate lengthy bureaucratic systems. One interview respondent said, *“Fines are so low that industry would rather pay the annual fine than the cost to upgrade equipment to fortify.”*

Respondents from both sectors believed that credibility and perceived effectiveness of regulatory bodies comes largely from consistent follow-though on enforcement measures, regular and unannounced inspections, and acting in a fair and transparent manner with all industrial entities. This also includes monitoring at the most appropriate place for enforcement. As one questionnaire respondent said, *“At the retail level, their point is, ‘Why are we to be penalized instead of factories?’”*

Perceived effectiveness of regulatory agencies comes from consistent follow-through on enforcement measures and transparency with industry.

Thirteen of 36 questionnaire respondents (36%) reported that factory inspections are announced beforehand. Even if visits are not announced, one questionnaire respondent noted, *“[News of inspection] spreads in advance by local people, media, etc. Once visits start in one factory, often surrounding factories quickly shut down and hide operations to avoid regulators from visiting.”*

Six of 18 questionnaire respondents from regulatory agencies (33%) believed that their regulatory system is robust and responds quickly to non-compliance. When asked about data management, 10 of 16 regulatory agency respondents (63%) reported that data is well managed or translated into action to improve compliance, and 9 respondents (56%) reported sharing their data regularly with stakeholders.

Another emerging theme from interview and questionnaire responses was that attitudes of respect and trust are lacking between the sectors. Of the 20 industry respondents, 12 (60%) reported that regulatory agencies do not believe industry puts effort into producing quality fortified food, and the same number reported they do not believe regulatory agencies do their job fairly. Seven of 20 industry respondents (35%) reported having a poor working relationship with their counterparts in regulatory agencies. As one interviewee suggested, “*Good governance calls for well-trained, motivated food inspectors who make an effort to be constructive, cooperative, and helpful, rather than having an attitude of policing, only trying to find what’s wrong.”*

### Prioritized Human and Financial Resources

Most questionnaire respondents (14 of 17, or 82%) from regulatory agencies noted their current funding is not completely sustainable over the next 5 years, and 16 of 18 respondents (89%) felt national budget allocations are the most stable source of funding necessary for regulatory success. Some respondents have detailed success stories in stretching their limited budgets. Of the 18 questionnaire respondents from regulatory agencies, 8 mentioned introducing a network of local offices that saves on inspector travel costs; 6 mentioned generating incomes through inspection, licensing, and testing services; and 5 cited the use of civil society, consumer, or industry groups to assist with monitoring activities. Respondents echoed the importance of securing a sustainable budget for general inspections and food control first, allowing for a smooth introduction of fortification regulation into the system later.

A recurring theme among key informant government interviewees was that limited budget allocations resulted in low compensation and few opportunities for professional training, leading to high staff turnover. Of 16 regulatory agency respondents, 2 reported inspectors never receive a travel budget; overall, half reported inspectors received one on an irregular basis (less than 40% of the time). Interview respondents noted that workloads are taxing for regulatory agency staff who must prioritize food safety issues above those of quality and fortification. This was also a common theme among questionnaire respondents. One noted, *“[There is] pressure on limited human resources, equipment, and consumables to allocate … among competing regulatory priorities.”*

**FIGURE 5:**
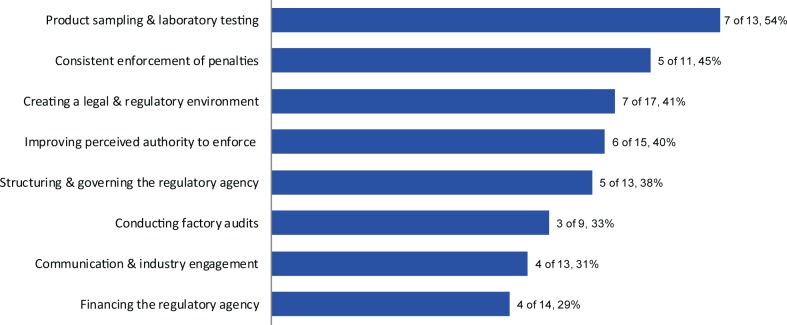
Regulatory Monitoring Areas Lacking a Trained Cadre of Regulatory Inspectors and Analysts, According to Open-Ended Responses From Regulatory Agency Respondents About Key Challenges^a^ ^a^ The analysis was based upon the responses of 18 respondents from 15 countries. A range of 9 to 17 respondents provided answers in each regulatory monitoring area.

Of 18 questionnaire respondents from regulatory agencies, 13 (72%) agreed that more inspectors were needed to effectively distribute the workload, and 15 of 17 (88%) believed that compliance at the industry level would improve with greater monitoring frequency. Thus, regulatory agency respondents believed they should be doing more, but they lacked financial and human resources. A recurring theme throughout the interviews and surveys was that lack of technically trained staff prevented regulatory agencies from being more effective overall. This was pervasive along the continuum of regulatory monitoring and was discussed in questions on a variety of topics (Figure 5).

Regulatory agency respondents believed they should be doing more but lacked the necessary financial and human resources.

Finally, the importance of an adequately resourced laboratory was emphasized by respondents. Of the 16 regulatory agency respondents, 6 (38%) reported a lack of equipment and inputs for laboratories while a lack of trained staff and technical capacity were reported by 8 respondents (50%). Interview respondents noted that the lack of laboratory capacity slowed the testing process and prohibited inspectors from making cost-efficient judgments about required follow-up action. Even where resources and trained staff were available, regulatory agencies faced additional challenges. As one respondent said, *“The greatest challenge is getting test results acted upon for compliance.”*

## DISCUSSION

In this paper, we highlighted the barriers and successes experienced by professionals working in regulatory monitoring and industries that fortify foods. Our methodology’s strength in eliciting rich qualitative descriptions of respondents’ perspectives enabled us to identify barriers and good practices in regulatory monitoring, classifying them into themes for further discussion. Insufficient and inconsistent monitoring persists because of perceived low risk of detection for non-fortified and underfortified foods. These perceptions can be due to actual resource and capacity constraints within regulatory agencies and unclear legislation, but as many of the respondents described, a major contributor is a sense of political unwillingness.

Attitudes toward established food safety laws and compliance with them based on the probability of detection and prosecution has been explored previously; parallels can be drawn to similar issues of food quality and fortification.^34^^,^^35^ Some businesses are “political citizens” who comply with regulations unless they consider rules unreasonable; some are “economic actors” who comply when it is profitable; and some are “incompetent organizations” who are willing to comply but are not well enough equipped or knowledgeable to do so.^36^ The first attitude can be found among food producers who attempt to fortify but are non-compliant due to challenges in technical or laboratory capacity. The second is prevalent among food producers in contexts where there is competition with non-fortifying producers or imports and where access to affordable premix is lacking. The third attitude is most often found in countries just beginning to fortify, where a lack of knowledge or clarity in the regulations must be overcome. As will be echoed throughout, regulatory agencies could improve their ability to communicate with industry to fully understand how to engage them and meet compliance targets.

**FIGURE 6:**
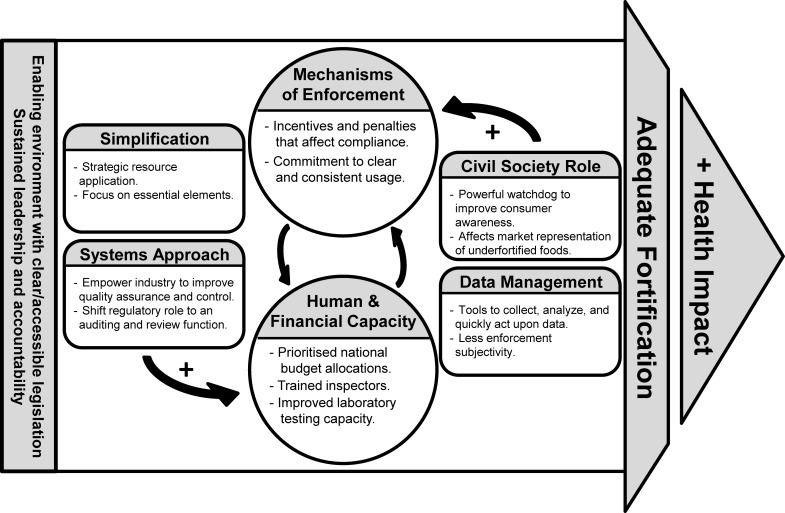
Relationship Between Regulatory Monitoring Elements in Ensuring Food Vehicles Are Adequately Fortified and Can Contribute to a Positive Health Impact

Figure 6 depicts the relationship between regulatory monitoring elements and a positive impact on health. Enforcement mechanisms and human and financial resource capacity form a strong feedback loop. In many regulatory monitoring systems, budget constraints lead to insufficient resources devoted to fortification regulation, often because of competing priorities. Low enforcement and compliance result largely from a lack of resources or an unwillingness to detect underfortified foods and hold industry accountable. Without strong political willingness to enforce, justifying investments in resources for enforcement is difficult. Injections of positive interventions into this cycle, such as using a systems approach, simplifying data collection and management, and increasing the role of civil society, can help to decrease the strain on resources. There is a need for an enabling environment to underpin technical enforcement capacity with legal and political commitment and willingness. This includes clear legislation and sustained leadership and accountability in both the private and public sectors. The schematic is further described in the following sections.

### Food Law and Legislative Environment

Food laws, regulations, and standards related to mandatory fortification are frequently fragmented and do not clearly present the roles of stakeholders or the array of enforcement mechanisms that can be used legally. Many interview and questionnaire respondents described clear and consolidated legislation as a prioritized step in improving compliance with national fortification regulations. Clear laws and regulations can foster an enabling environment and good working relationships between producers and regulatory agencies. Requiring fortification through mandatory legal instruments has been a recommended strategy to level the playing field, incentivizing industry to fortify by removing competition from non-fortifying producers and providing the basis for consistent legal enforcement.^37^ When the mandate is not enforced, industries may choose to stop fortifying to increase profits, jeopardizing the nutritional impact on the population.

Mandatory legal instruments requiring food fortification may help level the playing field for industry.

### Mechanisms of Regulation Enforcement

One of the most critical themes drawn from this study’s findings, especially from the perspective of industry, is that enforcement is an important driver of compliance with national standards. At the root of underfortified products is insufficient regulatory monitoring and enforcement that lead to non-compliance among industries.^38^^-^^40^ Industry sees regulatory monitoring as related to how often and how well regulatory agencies inspect facilities and consistently implement enforcement measures. Moral authority can be garnered through perceptions of effectiveness and coordination, which are linked to agency structure, governance, transparency in decision making, and funding prioritization.^41^

Insufficient regulatory monitoring and enforcement is at the root of underfortified products.

While most respondents reported using some combination of incentives and penalties for enforcement, it is their effectiveness in strength and application that matter most. Regulation on paper will not improve fortification compliance without incentives and consequences that are real, strong enough to drive underfortified foods out of markets and production facilities, and consistently applied. Good enforcement requires the capacity of regulatory agencies and their public laboratories to detect and act upon non-compliance.

Regulation on paper will not improve fortification compliance alone.

Claiming that enforcement of penalties presents political risk was a common theme among respondents and has been previously encountered.^20^^,^^42^ To combat this, regulatory agencies must show proper leadership and communicate with industry to ensure the reasons for regulations and penalties are understood as useful in leveling the playing field for industry.

### Human and Financial Resources

Funding is a critical element of food control management and governance, providing the resources necessary for action and a sense of government prioritization. Food safety is typically prioritized more highly than food quality, including fortification, because food safety issues generally present a higher and more immediate risk than an issue of sub-standard quality.^24^ A few cases of *E. coli* are a more immediate threat to public health than the long-term, cumulative health consequences of micronutrient deficiencies, even though the detrimental effects of nutrient deficiencies, such as poor cognitive development, immunity, and productivity, affect a greater proportion of the population.^29^^,^^43^

Without a stable funding source, countries must cope with uncertainty in their budgets, impacting the quality of their regulatory capabilities. The effectiveness of inspection and verification services relies on qualified, trained, efficient, and honest food inspectors who are able to collect samples for laboratory testing and carry out quality and safety evaluations.^44^^,^^45^ Questionnaire data reveal that many countries lack adequate public-sector laboratory capacity, including equipment, supplies, and trained personnel. Sampling quantity and data quality are likely to reflect the quality of technical and human resources available within laboratories.

Regulatory agencies are more likely to succeed with a core team of trained inspectors who will remain in the position long-term, which may require incentives and other motivational elements such as travel budgets and recognition for timely results. Such a workforce must be accompanied by an operating environment that places a strong emphasis on food quality, inspector honesty and integrity in reporting, and communicating with industry to remedy issues of overall quality control. Inspectors also need to understand the relevant food laws and regulations, including methods they can legally use to inspect industry facilities and hold producers accountable for the findings.

Industry also has a role to play in enacting QA/QC measures and consistently producing foods that are compliant against mandated national standards. To address overall food quality, industry capacity must be improved in using good manufacturing practices and working with regulatory agency counterparts to remedy issues of access and affordability of inputs and premix.

### Increasing the Role of Civil Society

Fortified foods are considered credence goods, those that consumers cannot easily evaluate in order to demand a higher quality. Fortified and non-fortified products are virtually identical and without the use of some form of analytical equipment, consumers have little indication as to whether vitamins and minerals have been added in the declared amounts or will perform as claimed. They must take the stated claims of manufacturers on faith. This same information asymmetry can also describe the relationship between fortified food producers and their micronutrient premix suppliers.

For some credence goods, including fortified foods, product demand depends largely on branding and marketing to provide consumers with a recognizable way to distinguish between products.^46^ Since consumers are easily cheated into paying higher prices for claims of higher-quality products, there is little market incentive for food producers to invest in improvements to increase the quality of their products. Food producers who wish to pawn off lower-quality goods as higher ones will therefore drive out legitimate business.^47^ The burden for increasing incentives to invest in fortification (and food quality more broadly), therefore, largely falls on regulatory agencies.

As success stories from respondents detail, regulatory agencies have benefited from working with civil society organizations, including industry and consumer associations. There have been documented successes where village health committees helped to monitor small and local retailers.^48^ Civil society can be a powerful watchdog, improving consumer awareness of those food producers that pass off their underfortified products as good consumer choices. Civil society can also be an important regulatory assistant, lessening the financial and workload burden of the national regulatory offices as a stopgap measure until local offices can be built and properly staffed.^44^^,^^49^

Civil society can be a powerful watchdog by improving consumer awareness of food producers that underfortify.

### Simplifying Data Collection and Management

It is advantageous to streamline workflows and apply resources strategically to essential elements. Even with a consolidated wheat milling sector in a small geographic area, Jordan’s regulatory inspectors faced difficulties in their ability to conduct on-site surveillance and monitoring on a regular basis. To overcome this, a simple external monitoring system was adopted that collects 3 indicators (monthly production of wheat flour, number of boxes of premix used, and iron concentration in a flour sample) that can be easily analyzed and used to make programmatic decisions by a low-resource regulatory agency.^50^ Similarly, Egypt’s fortified *Baladi* bread producers introduced an online fortification monitoring system in 2011 that generates automated alerts in cases of outliers from the normal fortification range, shortages in warehoused premix stock, and other system disturbances that may result in underfortified products.^51^ Just 2 years after implementation, the latest compliance data claim that 95% of Egypt’s flour is adequately fortified to national standards.^51^ Finally, Zimbabwe’s Ministry of Health and Child Care is in the process of incorporating key fortification monitoring indicators into their District Health Information Software. This allows for the efficient and consistent tracking of monitoring data that can be disaggregated by location, date, brand, or producer through an already-existing centralized data capture system (personal communication with Arthur Pagiwa, Zimbabwe Country Coordinator, Project Healthy Children, Jul 2015).

Integrating key fortification indicators into existing health and nutrition monitoring systems helps with efficient and consistent tracking of compliance.

Inspections are most appropriately conducted at the point of production and/or import, where it is most efficient and affordable to remediate underfortification.^52^ Monitoring at retail outlets may have a role in creating awareness among retailers and consumers or as verification of nutrient retention, but it is ineffective as an enforcement tool since it is often difficult to trace non-compliant products back to their production or import source.^20^

### A Systems Approach to Monitoring

Many countries have focused solely on laboratory testing of final product samples for conformance with national standards; however, this strategy is costly.^20^^,^^29^ Furthermore, final product sampling and analysis techniques have large margins of error for some micronutrients, random samples are not always a true reflection of overall performance, and the turnaround of information is often too slow to make timely modifications. A “systems” approach to monitoring facilitates preventive measures at all stages of the food value chain so that underfortified products can be identified and remedied earlier along the chain. This approach, which is in conjunction with the principles of Good Manufacturing Practices (GMP) and Hazard Analysis/Quality Analysis and Critical Control Points (HACCP/QACCP), has been the industry standard in many countries for the manufacture of pharmaceuticals and, in many cases, processed and fortified foods, for over a decade.^24^^,^^53^^-^^56^ This approach facilitates industry to keep good records and entrusts them with the primary responsibility for safety and quality, leaving regulatory inspections to verify whether industrial entities have the adequate raw materials, equipment, systems, and procedures in place for the manufacturing processes to result in consistent production of adequately fortified foods. Testing product samples is still required and is critical but relegated to a validation role.^24^^,^^53^

Using the systems approach requires a cooperative working relationship between regulatory agencies and food producers with a mutual understanding that sustained violation will be addressed.^37^^,^^57^^,^^58^ Effective inspections also require a platform for information exchange to build cooperation and trust. Success stories have proven that the systems approach does have broad applicability and relevance to developing countries.^50^^,^^59^ Codes of practice that delineate monitoring activities using the systems approach have been drafted and are awaiting approval in South Africa,^60^ Zimbabwe,^61^ and Mozambique, and the idea is gaining traction elsewhere in Africa and Asia (personal communication with Philip Randall, Director, PCubed, Jul 2015). Many questionnaire respondents in our study agreed that it is a better use of scarce resources, although there are a number of constraints along the food value chain that present a barrier to this approach, including a lack of trust between government inspectors and industry personnel, a lack of training among industry staff, and a lack of standard operating procedures that prevent consistent recordkeeping. More operational research is needed in this area to further develop the systems approach for regulatory monitoring of fortified foods, specifically in the context of low- and middle-income countries in Africa and Asia.

A systems approach to monitoring requires a cooperative working relationship between regulatory agencies and food industry.

### Recommendations for Policy and Practice

Taking into consideration lessons learned from food safety control^34^^,^^35^ and pharmaceutical safety and quality control,^42^^,^^44^^,^^45^ 7 broad-reaching recommendations for improving fortification compliance can be synthesized from this study:

1. **Legislation:** Develop and implement clear legislation that outlines roles and responsibilities of all stakeholders, provides an enabling environment within the private and public sectors, and includes applicable enforcement mechanisms.
2. **Leadership:** Identify strong leadership within government that facilitates the prioritization of fortification programming and subsequent enforcement and national budget allocations.
3. **Enforcement:** Focus on strong, effective enforcement mechanisms that influence compliance with national standards. Encourage leadership to consistently use enforcement mechanisms to hold industry accountable.
4. **Financial and Human Capacity:** Improve capacity at the regulatory agency and industry levels. Prioritize funding for inspector training, sample collection and laboratory testing, and technology transfer to industry.
5. **Community:** Engage civil society and community organizations as a third-party to build consumer support and knowledge and to reduce the regulatory resource burden.
6. **Data Capture:** Simplify regulatory monitoring management processes, including streamlined data collection and feedback mechanisms for action.
7. **Relationship Building:** Build relationships and trust with industry counterparts so the systems approach can be an achievable goal.

Sustained government funding, in addition to external funding, is required to improve these critical areas of regulatory monitoring. While questionnaire respondents stressed the importance of national budget allocations to ensure funding sustainability, international donors must respond to this call to action to provide the impetus for countries as they build capacity, improve systems, and allocate additional funds from national budgets. Recommendations should be made by donors and technical assistance groups on which regional agencies and experts to use for adequate inspector trainings. Public-private partnerships must also be motivated and leveraged as key drivers of capacity strengthening, trust building, and funding for continued improvements at both industry and regulatory agency levels.

Public-private partnerships must be leveraged as key drivers of capacity strengthening, trust building, and funding for continued improvements.

### Limitations

This assessment is not without limitations that must be considered. Selection bias may have been introduced since respondents may have been better informed or may consider fortification efforts more important than non-respondents. Thus, if those respondents that consider fortification important agree that more attention and resources must be directed toward regulatory monitoring, the authors assume the issues surrounding poor monitoring are similar or worse for those countries where fortification efforts are not prioritized. Data analysis did not distinguish between food vehicles or the year that fortification was mandated. There is the possibility that monitoring processes are different for each food vehicle, due to differences in industry structure and consolidation, while regulatory monitoring is likely to improve over time as experience and knowledge increase.

We anticipated a degree of social desirability bias to occur, especially on the part of regulatory agencies overstating their capacity and monitoring activities. This calls into question the validity of their responses, although it likely underestimates the prevalence of discussed behaviors, making the recommendations even more pertinent. Industry respondents were mostly from larger corporations and come from the perspective of 1 vehicle, whereas regulatory monitoring respondents may answer from the perspective of up to 5 vehicles that are mandated within their country and covering the range of industry sizes. Some findings may not be relevant for small- and medium-sized producers that operate under different constraints and monitoring contexts.

The sample size was relatively small; thus, this study intended to summarize perspectives and experiences from a qualitative point of view while providing an inference that the quality of fortified foods and the systems in place through which they are monitored needs improvement, particularly in low- and middle-income African and Asian countries. The study does not attempt to present a comprehensive quantitative survey of fortification program compliance globally. Nor does it claim to provide evidence-based solutions to improve compliance of fortification programs. Further research and independent evaluations will be required to do this. The authors hope this manuscript will provide a call to action for independent researchers to initiate critical evaluations exploring specific areas of need in greater depth. The chosen methodology of the questionnaire dissemination, which relied upon country staff from GAIN and PHC to assist respondents, was designed to elicit a higher response quality, rather than quantity, especially since the technical subject matter may have affected question comprehension.

## CONCLUSION

Regulatory monitoring of food fortification is a complex process requiring leadership, good governance, and coordination. Mandatory legislation will not automatically lead to increased coverage of fortified products without proper enforcement and adequate capacity; likewise, focusing investment on upgrading technical skills and facilities will not automatically lead to the feedback mechanisms necessary to identify and recall underfortified products. Only by taking concrete steps to improve the entire regulatory system will a nutrition strategy that uses fortification see its intended health effects.

Challenges to enabling fortification compliance include economic disincentives at the industry level and a lack of prioritization and perceptions of political risk around enforcement at the government level. Investment strategies should focus on strong and consistent enforcement mechanisms that include a well-trained cadre of food inspectors, quality laboratories, clear legal instruments, simplified data capture mechanisms, and the use of civil society, all underpinned by strong government leadership. By improving these components of implementation, detection and prosecution of underfortified foods will improve and fortification programs will reach beyond the current 45% coverage of adequately fortified foods to attain their intended health impact.

## Notes

**Peer Reviewed**
